# Preliminary evaluation of FAPI-04-PET/CT for differentiating recurrence and post-treatment changes in high-grade gliomas

**DOI:** 10.37349/etat.2024.00276

**Published:** 2024-10-31

**Authors:** Indraja D. Dev, Ameya D. Puranik, Venkatesh Rangarajan, Sukriti Patra, Nilendu Purandare, Arpita Sahu, Amitkumar Choudhary, Kajari Bhattacharya, Tejpal Gupta, Abhishek Chatterjee, Archya Dasgupta, Aliasgar Moiyadi, Prakash Shetty, Vikas Singh, Epari Sridhar, Ayushi Sahay, Aekta Shah, Suchismita Ghosh, Sayak Choudhury, Sneha Shah, Archi Agrawal

**Affiliations:** National Cancer Center/Cancer Hospital, Chinese Academy of Medical Sciences, China; ^1^Depts of Nuclear Medicine and Molecular Imaging, Tata Memorial Hospital and Advanced Center for Treatment, Research and Education in Cancer (ACTREC), Homi Bhabha National University, Dr E Borges Marg, Parel, Mumbai 400012, India; ^2^Radiodiagnosis, Tata Memorial Hospital and Advanced Center for Treatment, Research and Education in Cancer (ACTREC), Homi Bhabha National University, Dr E Borges Marg, Parel, Mumbai 400012, India; ^3^Radiation Oncology, Tata Memorial Hospital and Advanced Center for Treatment, Research and Education in Cancer (ACTREC), Homi Bhabha National University, Dr E Borges Marg, Parel, Mumbai 400012, India; ^4^Neurosurgery, Tata Memorial Hospital and Advanced Center for Treatment, Research and Education in Cancer (ACTREC), Homi Bhabha National University, Dr E Borges Marg, Parel, Mumbai 400012, India; ^5^Pathology, Tata Memorial Hospital and Advanced Center for Treatment, Research and Education in Cancer (ACTREC), Homi Bhabha National University, Dr E Borges Marg, Parel, Mumbai 400012, India

**Keywords:** FAPI-04, fluoro-ethyl tyrosine, glioma, PET

## Abstract

Fibroblast-activated protein (FAP) expression in glial cells is attributed to FAP-positive foci on tumor vessels and neoplastic cells. Preclinical and pilot studies have shown FAP expression in high-grade gliomas. We aimed at comparing PET imaging with FAP-inhibitor (FAPI-PET) with current standard, i.e., fluoro-ethyl tyrosine (FET) PET in post-treatment setting to differentiate recurrence and post-treatment changes. 6 patients with WHO Grade III and IV glioma who received standard treatment underwent Ga-68-FAPI-04 PET/CT (FAPI-PET/CT). Tracer uptake greater than background was considered positive. FET PET was performed and interpreted as per institutional standards, which formed the basis of treatment decision. There was concordance between FAPI expression and FET uptake in 5 patients suggestive of disease recurrence. There was no FAPI expression seen in 1 patient, in whom FET PET was suggestive of post-treatment changes. FAPI PET uptake correlated with amino acid expression to differentiate post treatment changes from recurrence in high-grade glial tumors; further validation with prospective study and histopathological confirmation is needed.

## Introduction

The “holy grail” in brain tumor imaging is differentiating post-treatment changes from tumor recurrence, and fluoro-ethyl tyrosine (FET) PET has addressed this issue to some extent [1, 2]. While magnetic resonance imaging (MRI) continues to remain the gold standard for this indication, there is a unmet need for a functional imaging tracer which addition to providing a diagnosis like FET PET, in addition would also have a theranostic dimension. Also, considering the variability in amino acid expression in select cases, there is scope for a novel tracer with more physiological localisation to step-in [3, 4]. The natural pathway of glial cell progression is via “local” infiltration, which shifts the focus on local tumor micro-environment (TME). This includes surrounding blood vessels, immune cells, fibroblasts, signaling molecules and the extracellular matrix (ECM). Animal studies have shown that fibroblast-activated protein (FAP) is over-expressed in vitro and in situ in glioblastoma (GBM) cells and tumor stroma especially in proximity to blood vessels [5]. FAP has also shown a tendency to further tumor infiltration through the brain ECM as well as secretion of pro-angiogenic factors and TGF-beta [6]. A quinoline-based FAP-specific inhibitor [7], has been found suitable for labeling with radio-isotope (Gallium-68) using a chelator and has led to development of a new class of radiopharmaceuticals which are highly promising as molecular imaging probe—Ga-68-FAPI-04. FAPI PET specifically binds to sites where there is cancer-associated fibroblastic proliferation. We therefore conducted a comparative assessment of Ga-68-FAPI-04 PET/CT (FAP PET) with existing standard of care, i.e., FET PET in patients with WHO Grade III and IV glioma.

## Materials and methods

We studied a retrospective cohort of six patients of higher grade (WHO Grade III and IV) glioma, which underwent surgery followed by radiotherapy and temozolamide (TMZ) in concurrent setting. Patient characteristics are described in Table 1. MRI of brain was performed in all patients with inclusion of standard and advanced sequences like diffusion-weighted imaging (DWI), spectroscopy and perfusion imaging. Multi-disciplinary clinic decision was to perform FET PET for differentiating recurrence from post-treatment changes, as per institutional protocol. Based on approval by Institutional Review Board for a pilot study, these patients underwent FAPI PET. Informed consent to participate in the study was obtained from all participants. Final decision regarding the diagnosis was taken by reviewing FET PET, FAPI PET and serial MRI images in a multidisciplinary clinic. Details of imaging findings, diagnosis and follow-up are mentioned in Table 2.

**Table 1:** Patient characteristics

**Patient No**	**Age**	**Gender**	**WHO Grade**	**Histopathology**	**IDH mutation status**	**Primary treatment received**
1	35	Male	III	Oligodendroglioma	Mutant	Surgery, concurrent CT/RT
2	66	Male	IV	GBM	Wild-type	Surgery, concurrent CT/RT, adjuvant TMZ
3	39	Female	IV	GBM	Mutant	Surgery, concurrent CTRT, redo surgery, adjuvant TMZ
4	52	Female	IV	GBM	Non contributory	Surgery, concurrent CTRT, adjuvant TMZ
5	42	Male	III	Astrocytoma	Mutant	Surgery, concurrent CTRT
6	47	Male	IV	GBM	Wild-type	Surgery, concurrent CTRT, adjuvant TMZ

CT/RT: chemotherapy and radiation therapy; TMZ: temozolamide; GBM: glioblastoma

**Table 2:** FAPI and FET PET imaging interpretation & follow up data

**Patient**	**MRI findings**			FET*** PET	**FAPI PET**	**Diagnosis**	**Follow-up**
	**T1 post-contrast**	**Perfusion**	**BT-RADS**				
1	Nodular enhancement	Hyper	3C**	2.9	Positive	Recurrence	Re-surgery, HPR—Grade IV GBM
2	Nodular enhancement	Hyper	3C	4.07	Positive	Recurrence	Salvage TMZ
3	Irregular enhancement “Swiss cheese” pattern	Iso	3B*	2.5	Negative	Post-treatment changes	Follow-up MRI—no change
4	Nodular enhancement	Hyper	3B	3.6	Positive	Recurrence	Salvage TMZ
5	Nodular enhancement	Iso	3B	5.9	Positive	Recurrence	On Bevacizumab
6	Nodular enhancement “Swiss cheese” pattern	Hyper	3C	4.0	Positive	Recurrence	Salvage TMZ

BT-RADS 3B*: indeterminate mix of treatment effect and tumor progression [13]; BT-RADS 3C**: tumor progression favored [13]; FET PET***: tumor-to-white mater cut-off of 2.62 to differentiate between recurrence and post treatment changes [8]; HPR: histopathology report; GBM: glioblastoma; TMZ: temozolamide; MRI: magnetic resonance imaging; FAP: fibroblast-activated protein; FET: fluoro-ethyl tyrosine

### FET PET imaging and analysis

185-222 MBq (5-6 mCi) of F-18-FET was injected intravenously. Static imaging of the brain was performed at 20 min on Philips Gemini TF TOF 16/64 PET/CT scanners at 5 min per bed position. CT scan of the brain (120 kV, 250 mAs/slice, and thickness—3 mm) was acquired in the craniocaudal direction. Images were iteratively reconstructed in all the three planes. All images were reconstructed with an OSEM 2D algorithm (2 iterations, 21 subsets, 4-mm Gaussian post-reconstruction filter), corrected for attenuation, scatter, and radioactive decay, and displayed in a 256 × 256 matrix with 2.7 mm × 2.7 mm × 3.0 mm voxels. Tumor to contralateral white matter ratio was used as a semi-quantitative parameter for image interpretation with a cutoff of 2.62, as derived from our published institutional cohort of high-grade gliomas [8].

### FAPI preparation and image analysis

Gallium-68 was eluted from the Germanium-68/Gallium-68 generator system (ITM Gmbh, Germany). Labeling was performed in a semi-automated synthesis module using 33 micrograms of peptide, DOTA-FAPI-04, dissolved in 0.25 M sodium acetate buffer. Peptide was heated at 95°C for 5 min followed by elution of Ga-68. Peptide and eluate were heated for 10 min for 95°C and passed through C-18 cartridge preconditioned with 70% of ethanol and normal saline. Labeled product was passed through 0.22 µm filter. 185-222 MBq (5-6 mCi) of Ga-68-FAPI was injected intravenously and scans were acquired at 45 min. Dedicated static images of brain were acquired with similar acquisition parameters as for FET PET/CT. Qualitative visual assessment of the scans was done independently by two nuclear medicine physicians. FAPI PET was interpreted using visual parameters. Tracer uptake more than normal background uptake was interpreted as positive.

## Results

**Figure 1:**
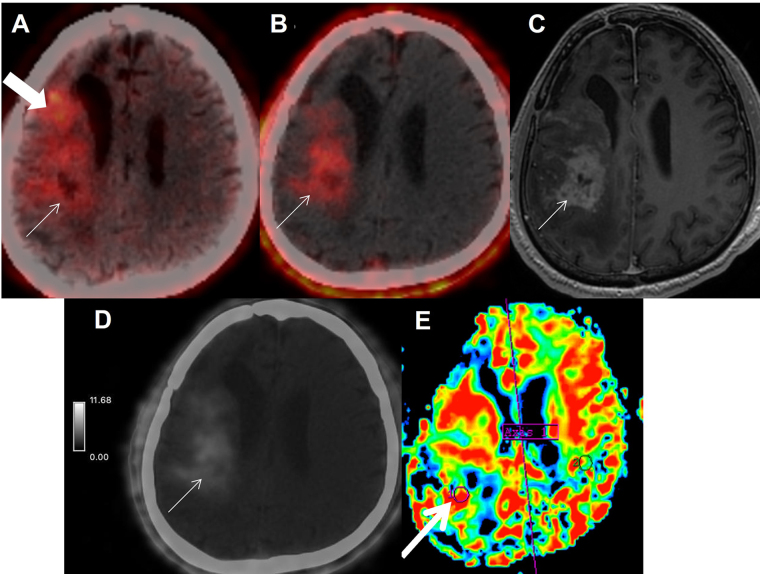
Patient 2 is a 66-year-old male, case of right temporal WHO Grade IV GBM, post surgery, radiation therapy with concurrent and adjuvant temozolamide (completed in January 2019), presented in June 2021 with new-onset seizures. MRI and FET PET was advised. (A) Axial FET PET showed tracer uptake in the right fronto-temporal and insular region (arrow), extending to anterior periventricular region (block-arrow). T-W ratio was 4.07 suggestive of disease recurrence. (B) Axial FAPI PET/CT image showed tracer uptake in the right fronto-temporal and insular region (arrow), with SUV_max_ of 2.8. It corresponded to the enhancing lesion on axial post contrast T-1 weighted image (C, arrow), which showed focal hyperperfusion (E-arrow) gray scale image of axial FAPI PET with intensity map (D, arrow) shows high-grade uptake at the site of MR and FET depicted recurrence. FET uptake was also seen in the residual disease component in the anterior periventricular region (block-arrow), which was not seen on FAPI PET/CT. MRI: magnetic resonance imaging; FET: fluoro-ethyl tyrosine; GBM: glioblastoma

**Figure 2:**
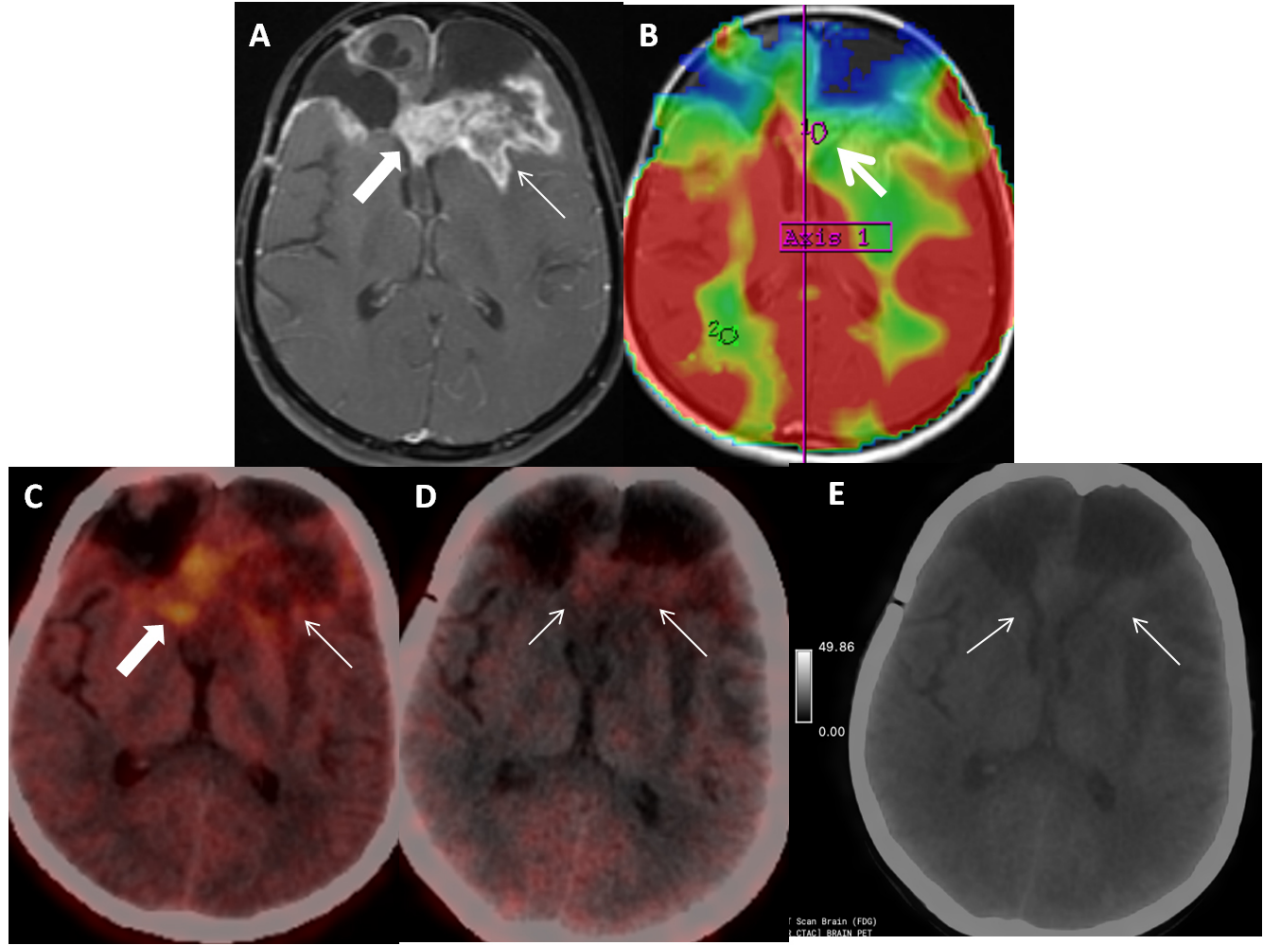
Patient 3 is a 39-year-old female, treated case of IDH-mutant WHO Grade IV glioma (last treatment in June 2019), recurrence on surveillance MRI (September 2021), underwent re-surgery, and received adjuvant temozolamide. Patient presented with seizures in December 2021. Axial post contrast T1-weighted image (A, arrow) shows predominantly feathery enhancement (arrow) with few areas of thick peripheral enhancement suggestive of Swiss cheese appearance (block arrow) and hypoperfusion (B, arrow). Correlative axial FET PET/CT image show tracer uptake (C, arrow) with T-W ratio of 2.5 suggestive of post treatment change. Axial FAPI PET/CT and gray scale FAPI PET image (D, E; arrows) shows low intensity diffuse tracer uptake in the corresponding region equal to background uptake interpreted as negative FAPI PET. Patient was asked to follow up with short interval MRI, which was unchanged thus confirming the diagnosis of post-treatment change. FET: fluoro-ethyl tyrosine; MRI: magnetic resonance imaging

**Figure 3:**
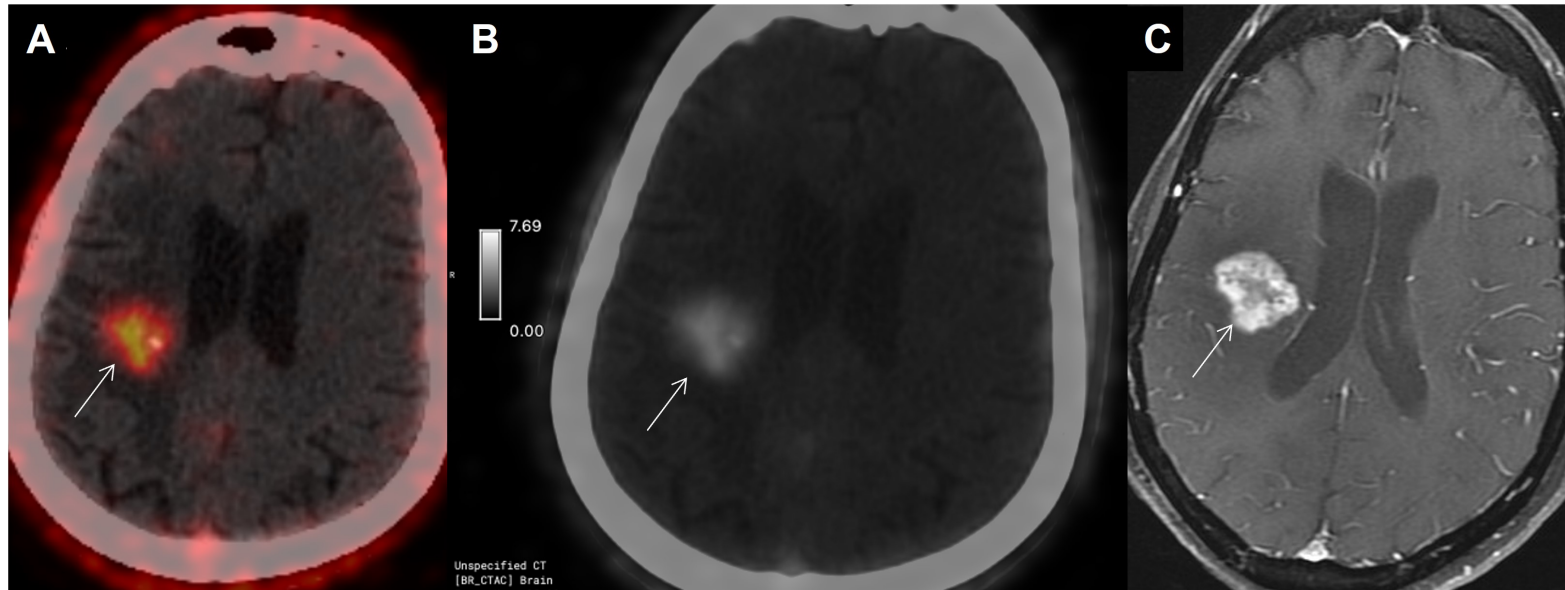
52-year-old case (patient 4), operated case of Grade IV GBM, post radiotherapy and temozolamide, index tumor was in left temporal region in November 2020. Now presented with left sided weakness. Imaging as advised with a clinico-radiological suspicion of post-treatment changes in the low-dose radiotherapy region in the opposite hemisphere. High intensity FAPI uptake was seen in right hippocampal region (more than background) on axial gray scale PET image (A, arrow), with SUV_max_ of 3.2; which matched the uptake on axial FET PET/CT (B, arrow) with T-W ratio of 3.6, suggestive of recurrence. Uptake pattern was concordant with enhancing lesion on axial post-contrast T1-weighted MR sequence (C, arrow) which was reported as BT-RADS 3B. Patient showed clinical worsening within 3 months and died. FET: fluoro-ethyl tyrosine; GBM: glioblastoma

**Figure 4:**
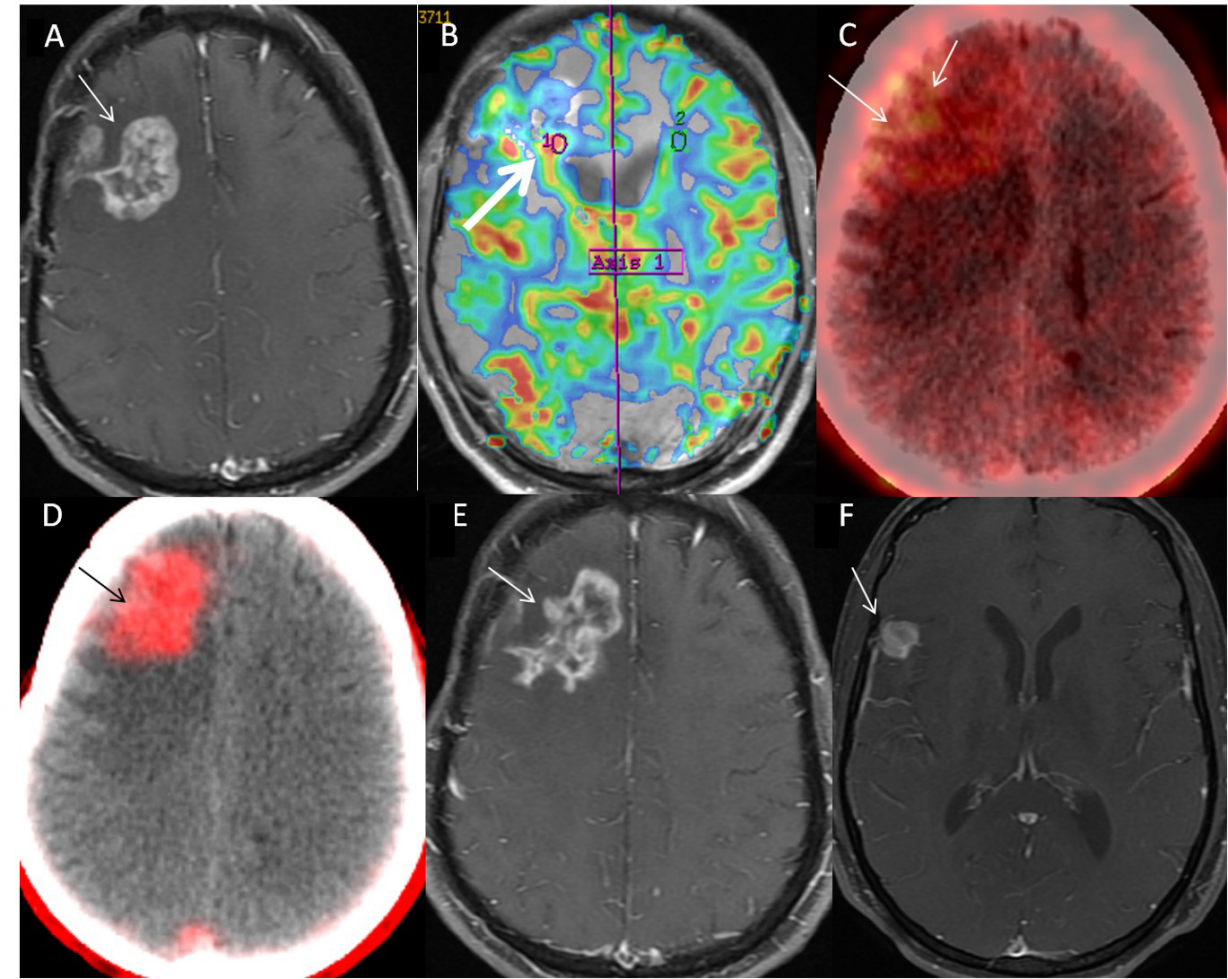
42-year-old male (patient 5) case of IDH-wild-type Grade IV glioma, of right fronto-parietal region, completed treatment in September 2021, presented with left-sided weakness in December 2021. Nodular enhancing lesion (Swiss cheese pattern) was seen on axial MR image (A, arrow), which showed a focal area of hyper perfusion with rCBV of 2.3 (B, arrow). This was attributed to predominantly post-treatment changes, BT-RADS 3B. FET PET shows patchy uptake in the lesion (C, arrow) with T-W of 4.0, despite the visual low-intensity of uptake. FAPI PET/CT image (D, arrow) showed well-defined area of intense uptake which corroborated with lesion on MR, with SUV_max_ of 3.6. Diagnosis of recurrence was established and patient was started on Bevacizumab. Follow-up MR at 3 months (E, arrow) showed mild reduction in extent of enhancement (pseudo-response to Bevacizumab) with a new enhancing lesion in right temporal region (F, arrow) suggestive of disease progression. rCBV: relative cerebral blood volume; FET: fluoro-ethyl tyrosine

Six patients with high grade glioma were studied. On histopathology, 4 had WHO Grade IV glioma (GBM) and 2 had WHO Grade III glioma; one being anaplastic astrocytoma and other anaplastic Grade III oligodendroglioma. All patients had received standard treatment, i.e., surgical debulking, followed by radiotherapy with concurrent TMZ. Clinical follow-up and/or MRI imaging was done as per routine practice. 3 patients complained of seizures, two developed mild hemiparesis, and one presented with headache. For the purpose of this study, enhancement pattern, perfusion and BT-RADS was considered on MRI. FET PET was performed. Five patients had T-W ratio more than 2.62 on FET PET, suggestive of recurrent disease (Figures 1, 2, and 3), with concordant uptake seen on FAPI PET. One patient (Figure 4) had T-W ratio less than 2.62 on FET PET which was suggestive of treatment related changes FAPI PET/CT revealed low grade uptake which was equal to background uptake, which matched the FET findings. These findings were confirmed either with histopathological diagnosis or serial imaging on subsequent follow up in a multidisciplinary joint clinic. All five patients with positive FET and FAPI findings were confirmed as having recurrent disease, of them, one patient underwent re-surgery, three patients were rechallenged with temozolomide and one patient was started on Bevacizumab. One patient with negative FET and FAPI PET findings was followed up with short interval MRI, which remained unchanged, thus suggestive of post-treatment changes (Figure 4).

## Discussion

The expression of FAP has on high-grade glial cells has been well-documented on in vitro studies performed using the human GBM cell lines, as well as on immunohistochemistry [9]. From a molecular imaging perspective, it is important to understand that FAP expression is elevated in the mesenchymal subtype of GBM, especially in transformed and stromal cells; this makes its utility more relevant in IDH-wild type and transformed IDH-mutant high-grade gliomas [10], which was seen in our series. Since amino-acid PET is the established standard for glioma-imaging [11], we compared FAPI PET with FET PET. Our study found a clear correlation between FAP and FET PET, for both recurrence and post-treatment changes. A head-to-head comparison of MRI and FAPI PET by Röhrich et al. [12], showed moderate correlation of relative cerebral blood volume (rCBV) with FAPI uptake when images were co-registered using software. However, it is important that the entire spectrum of MR findings and integrated diagnosis needs to be compared with FAPI uptake. In our institution, enhancement on post-contrast T-weighted image, BT-RADS (Brain Tumor Reporting and Data System) score [13] and MR perfusion (rCBV) were considered for decision-making. Multi-disciplinary clinic discussion prompted these patients to be referred for FET PET. L-amino-acid transporters (LAT-1) on glial cells form the basis of localisation of FET, whereas FAPI localizes to FAP which is present on tumor stroma especially in proximity to blood vessels. Low expression of LAT-1 has been reported in a subset of gliomas [14], leading to photopenia on FET PET, mechanism of which is unexplained. It is also seen that extent of FET uptake is more than the FAPI uptake (Figure 1); likely explanation being FET localisation in residual viable glial cells along with recurrent disease. In this regard, FAPI uptake is well-localised to area of recurrent disease seen on MR. This aspect is important in neuro-oncology imaging, wherein there is no definite surgical margin and differentiating between residual tumor, infiltrative component and recurrent disease often poses a challenge on conventional imaging. In addition, tumor delineation on FAPI PET can provide a robust gross tumor volume for radiotherapy planning, especially if FAPI volumes are fused with planning sequences of MR either with co-registration software or with a dedicated PET/R scanner. This is important especially since FET-based planning has not led to increment in survival, based on early phase II studies [15]. Considering the logistics and cost-effectiveness, generator-based production and labeling in hospital-based radiopharmacy makes FAPI PET a more cost-effective and easily available tracer for glioma imaging, unlike FET, which is produced at high costs in a cyclotron facility. From the theranostic perspective, positive expression on Ga-68 labeled FAPI imaging opens the door for therapy with its beta-emitting counterpart, i.e., lutetium (Lu-177)-labeled FAPI. Thus, emergence of FAPI PET has generated a fresh promise amongst patients with glial tumors, who after decades of advances in imaging and therapy have shown no increment in outcomes. The size of patient cohort is the limitation of this study, and hence we propose to design robust prospective trial using FAPI PET and FE PET in post-treatment assessment of glial tumors, which can help in establishing the exact potential of this novel tumor-targeting tracer.

We herewith uncover a novel target for imaging of post-treatment gliomas; and explore its utility in the molecular imaging landscape. It also has theranostic potential; with positive FAPI expression allows labeling with Lu-177 which can be used for radionuclide therapy.

## Abbreviations

FAP: fibroblast-activated protein
FAPI-PET: PET imaging with fibroblast-activated protein-inhibitor
FET: fluoro-ethyl tyrosine
GBM: glioblastoma
MRI: magnetic resonance imaging
rCBV: relative cerebral blood volume
TMZ: temozolamide

## Declarations

### Availability of data and materials

The raw data supporting the conclusions of this manuscript will be made available by the authors, without undue reservation, to any qualified researcher.

### Copyright

© The Author(s) 2024.
